# Characterizing the Type 6 Secretion System (T6SS) and its role in the virulence of avian pathogenic *Escherichia coli* strain APECO18

**DOI:** 10.7717/peerj.12631

**Published:** 2021-12-17

**Authors:** Aline L. de Oliveira, Nicolle L. Barbieri, Darby M. Newman, Meaghan M. Young, Lisa K. Nolan, Catherine M. Logue

**Affiliations:** 1 Department of Population Health, University of Georgia, Athens, GA, United States of America; 2 Department of Microbiology, University of Georgia, Athens, GA, United States of America; 3 Department of Infectious Diseases, University of Georgia, Athens, GA, United States of America

**Keywords:** Avian Pathogenic *Escherichia coli*, APEC, Type 6 secretion system, T6SS, Characterization, Virulence, Poultry, Disease

## Abstract

Avian pathogenic *E. coli* is the causative agent of extra-intestinal infections in birds known as colibacillosis, which can manifest as localized or systemic infections. The disease affects all stages of poultry production, resulting in economic losses that occur due to morbidity, carcass condemnation and increased mortality of the birds. APEC strains have a diverse virulence trait repertoire, which includes virulence factors involved in adherence to and invasion of the host cells, serum resistance factors, and toxins. However, the pathogenesis of APEC infections remains to be fully elucidated. The Type 6 secretion (T6SS) system has recently gained attention due to its role in the infection process and protection of bacteria from host defenses in human and animal pathogens. Previous work has shown that T6SS components are involved in the adherence to and invasion of host cells, as well as in the formation of biofilm, and intramacrophage bacterial replication. Here, we analyzed the frequency of T6SS genes *hcp*, *impK*, *evpB*, *vasK* and *icmF* in a collection of APEC strains and their potential role in virulence-associated phenotypes of APECO18. The T6SS genes were found to be significantly more prevalent in APEC than in fecal *E. coli* isolates from healthy birds. Expression of T6SS genes was analyzed in culture media and upon contact with host cells. Mutants were generated for *hcp*, *impK*, *evpB*, and *icmF* and characterized for their impact on virulence-associated phenotypes, including adherence to and invasion of host model cells, and resistance to predation by *Dictyostelium discoideum.* Deletion of the aforementioned genes did not significantly affect adherence and invasion capabilities of APECO18. Deletion of *hcp* reduced resistance of APECO18 to predation by *D. discoideum*, suggesting that T6SS is involved in the virulence of APECO18.

## Introduction

Avian pathogenic *Escherichia coli* causes extra-intestinal infections in birds known as colibacillosis, which can manifest as localized or systemic infections. Colibacillosis is most common in poultry but can also occur in other species of domestic and wild birds. The severity of the infection depends on the virulence traits of the strain, host status and predisposing factors (Barnes & Gross, 1997). The most common manifestations of APEC infections include cellulitis, a respiratory disease that frequently culminates in septicemia and lesions in organs such as liver, air sacs and heart; swollen head syndrome; omphalitis (inflammation of the yolk sac) and salpingitis (inflammation of the oviduct) in laying birds (Dho-Moulin & Fairbrother, 1999; Ngeleka et al., 1996; Nolan et al., 2020). Colibacillosis affects all stages of poultry production, resulting in economic losses due to morbidity, carcass condemnation and mortality of the birds (Dziva & Stevens, 2008).

Although the route of infection by APEC is not clearly defined, the oral and respiratory tracts seem to be the primary mode of entry (Harry & Hemsley, 1965). When the infection initiates in the upper respiratory tract it is probably due to the inhalation of contaminated dust. A high concentration of ammonia in the birds’ environment may be a factor that makes the birds prone to infection as it causes damage to the respiratory tract epithelium and favors the entry of bacteria via the respiratory tract. Other factors that may favor infection are exposure of the birds to high temperatures, limited space and insufficient disinfection of the environment (Dho-Moulin & Fairbrother, 1999). Even though several virulence factors that are involved in different stages of the infection process have been described for APEC, the pathogenesis of APEC strains remains unclear.

Secretion of proteins via protein machineries is recognized as a primary virulence property of bacteria, and Gram-negative bacteria have developed several secretion systems identified as types 1 through 6 (T1SS-T6SS) that transport proteins either into the extracellular milieu (T1SS, T2SS, T5SS) or directly into target cells (T3SS, T4SS, T6SS) (Costa et al., 2015). The Type 8 secretion system is dedicated to the secretion-assembly pathway known as nucleation-precipitation pathway, resulting in curli fiber production in Gram-negative bacteria including *E. coli* (Barnhart & Chapman, 2006; Hammar et al., 1995; Van Gerven et al., 2015). More recently, the Type 9 secretion system was identified in bacteroides and is involved in the pathogenesis of the Gram-negative periodontal pathogen *Porphyromonas gingivalis* (Sato et al., 2010). The Type 7 secretion system, also known as ESX, is found in the Gram-positive pathogen *Mycobacterium tuberculosis* and is specialized in secreting proteins across the complex cell envelope of mycobacteria (Abdallah et al., 2007). The Type 6 Secretion System (T6SS), Pukatzki et al. (2006) has gained attention due to its role in bacterial pathogenesis of Gram-negative organisms. The T6SS is composed of 13 conserved proteins that constitute the core of the system and a set of non-conserved proteins with regulatory and accessory functions (Boyer et al., 2009). These proteins assemble into a membrane complex, a baseplate, and a tail-like structure (comprised of the contractile sheath, inner tube and puncturing spike) (Cascales, 2008; Cascales & Cambillau, 2012; Planamente et al., 2016). Acting as a molecular syringe, the system targets both eukaryotic and prokaryotic cells, and its effectors play various roles on the host cell, including cytoskeleton rearrangement, cell invasion, and bacterial escape from the host defense mechanisms (Tseng, Tyler & Setubal, 2009).

The Type 6 secretion system is an atypical secretion system in which one protein may play a role as a structural component and secreted effector. This is the case of the best characterized protein in the system, Hcp (Hemolysin-coregulated protein) (Pukatzki, McAuley & Miyata, 2009).

The T6SS has been reported in a number of bacteria including the human and animal pathogen *Burkholderia mallei*, the fish pathogen *Edwardsiella tarda*, and the human pathogen *Pseudomonas fluorescens.* In each of these pathogens, the T6SS has been associated with the infection process, as well as protecting the bacteria from host defenses (Decoin et al., 2014; Schell et al., 2007; Zheng & Leung, 2007).

The role of T6SS in the pathogenesis of APEC was also demonstrated (De Pace et al., 2011; De Pace et al., 2010). The septicemia causing strain SEPT362 expresses a T6SS, and core components of the system Hcp, ClpV and IcmF are involved in biofilm formation, intramacrophage replication, *in vivo* virulence and in interaction with model host cells (De Pace et al., 2011; De Pace et al., 2010). A subsequent genome analysis showed that APEC may harbor up to three *loci* encoding T6SS and these vary in size and gene content (Ma et al., 2013). Another APEC strain, TW-XM, harbors two functional T6SS involved in different pathogenic pathways (Ma et al., 2014).

In the present study, we investigated the prevalence of genes from T6SS cluster 1 (T6SS1) and T6SS cluster 2 (T6SS2) in a collection of 454 APEC isolates and 102 poultry litter *E. coli* isolates, as well as 106 avian fecal *E. coli* (AFEC) isolates from healthy birds. We also investigated the role of T6SS in the pathogenesis of APECO18 by creating deletion mutants for *hcp*, *evpB*, *impK*, and *icmF*. The impact of these mutations on bacterial growth in minimal media was assessed. We also analyzed the impact of these genes on the ability of APECO18 to escape phagocytosis by using the alternative phagocytic cell model *Dictyostelium discoideum*, as well as the ability of APECO18 to adhere to and invade DF-1 chicken fibroblasts. Expression of T6SS genes by APECO18 in bacterial culture and upon contact with DF-1 chicken fibroblast cells was also evaluated with the goal of better understanding the role of the T6SS in APEC pathogenesis.

## Material and Methods

### Bacterial strains, plasmids and growth conditions

Strains and plasmids are shown in Table 1. The WT APEC strain was isolated from the pericardium of a chicken with signs of colisepticemia, and the sequenced genome is available at CP006830.1 (Nicholson et al., 2016). The O serogroup was identified at the *E. coli* reference center at Pennsylvania State University.

**Table 1:** Plasmids and strains used in this study.

**Plasmids**	**Genotype/description**	**Ref.**
pKD46	Lambda Red recombinase expression plasmid	Datsenko & Wanner (2000)
pKD3 Amp ^r^	template plasmid for FRT-flanked Amp cassette	Datsenko & Wanner (2000)
pKD3 Cm ^r^	template plasmid for FRT-flanked Cm cassette	Datsenko & Wanner (2000)
pCP20	FLP recombinase expression plasmid	Datsenko & Wanner (2000)
pBAD24	Cloning vector	Guzman et al. (1995)
pBAD24-*hcp*	pBAD24 with *hcp*	This work
pBAD24-*evpB*	pBAD24 with *evpB*	This work
pBAD24-*impK*	pBAD24 with *impK*	This work
pBAD24-*icmF*	pBAD24with *impG*	This work
**Strains**	**Genotype/description**	**Ref.**
*E. coli* DH5 *α*	F −Φ80lacZ ΔM15 Δ(lacZYA-argF) U169 recA1 endA1 hsdR17 (rK-, mK+) phoA supE44 *λ*–thi-1 gyrA96 relA1	Lab stock
APECO18 (380)	APECO18 WT	Lab stock
APEC Δ*hcp*	APECO18 with *hcp* deleted by *λ* red rec.	This work
APEC Δ*evpB*	APECO18 with *evpB* deleted by *λ* red rec.	This work
APEC Δ*impK*	APECO18 with *impK* deleted by *λ* red rec.	This work
APEC Δ*icmF*	APECO18 with *icmF* deleted by *λ* red rec.	This work
APEC Δ*hcp-* p*hcp*	APEC Δ*hcp* with *hcp* cloned into pBAD24	This work
APEC Δ*evpB-* p*evpB*	APEC Δ*evpB* with *evpB* cloned into pBAD24	This work
APEC Δ*impK-* p*impK*	APEC Δ*impK* with *impK* cloned into pBAD24	This work
APEC Δ*icmF-* p*icmF*	APEC Δ*icmF* with *icmF* cloned into pBAD24	This work

*E. coli* DH5*α* was used for cloning. All *E. coli* strains were grown in Lysogeny (LB) broth (BD Difco™, Franklin Lakes, NJ) at 37 °C with shaking, unless otherwise specified. The medium was supplemented with ampicillin (Amp 100 µg/mL), chloramphenicol (Cm 10–25 µg/mL) or L-arabinose (6.5 mM) as necessary.

### DNA extraction

Bacterial DNA was obtained from whole organisms using the boil prep method as previously described (De Oliveira et al., 2020). Specifically, cultures and cell lysate were centrifuged at 16,700 *g* for 3 min.

### Detection of T6SS genes by PCR

The presence of T6SS genes *hcp*, *evpB*, *impK*, *vasK* and *icmF* was analyzed in a collection of 106 avian fecal *E. coli* (AFEC) recovered from the feces of healthy production birds, 102 poultry litter *E. coli* isolated from the litter of poultry barns and 454 APEC isolates recovered from the lesions of production birds diagnosed with colibacillosis by polymerase chain reaction (PCR) amplification (see Table S1). APECO18 (CP006830.1) was used as template for primer design. Primers were obtained from Sigma (St. Louis, MO). Reactions were performed in a 25 µl volume as previously described (De Oliveira et al., 2020). The PCR conditions were as follows: 94 °C for 5 min; 30 cycles of 94 °C for 30 s, 60 °C for 30 s, 68 °C for 3 min, and a final extension step of 72 °C for 10 min. Primers used are listed in Table 2. APEC O18 was used as a positive control for primer check and sterile water in place of DNA for negative control check.

**Table 2:** Primers used in this study.

**Primer**	**Sequence (5′–3′)**	**Ref.**
	**Screening of T6SS genes**	
*hcp* _Fw	cgaaggtagcatcgaagtgg	This work
*hcp* _Rv	ttaaactccgccgttttcag	This work
*evpB* _Fw	agcgattcacgttctgcttt	This work
*evpB* _Rv	tgcacacaccagccattatt	This work
*impK* _Fw	ccactccagtcgcatttctt	This work
*impK* _Rv	attcagccagtggtgtagcc	This work
*icmF* _Fw	acaacgaggcggtaaaacag	This work
*icmF* _Rv	atgtaacgaacggcttccac	This work
	**Gene deletion**	
*hcp* Del_F:	atggctattcctgcttatctctggctgaaagatgacggcggcgcggatat^a^	This work
*hcp* Del_R:	tcaggcggaaggacgctcattccacgagtcggaatgaatgatgttgccgt^b^	This work
*evpB* Del_F	atgctgatgtctgtacaacaagaacattccacctctgaaactgcaacact^a^	This work
*evpB* Del_R	tcaggctttcgctttcggcatctgggaaaccagagaaaggttgatatcca^b^	This work
*impK* Del_F:	atgaaaaaagatatggatatcaatatcgatgcgctgctgcgcgacacgtt^a^	This work
*impK* Del_R:	ttaacgcaggctttgcggcagcagttcatccaccagtacattcagccagt^b^	This work
*icmF* Del_F:	gtgttcaaatttcccacatcccgactgttcagcacgttgaaatctgcgct^a^	This work
*icmF* Del_R:	ttaatacaacgtatccggtaaacggaacaggctgaacagaccgccggtga^b^	This work
	**Check deletion**	
*hcp* Check_F:	atcagtcttgttccgcgttc	This work
*hcp* Check_R:	tcaccagattgtgggtatgc	This work
*evpB* Check_F	tcagaactgcgtgatgaactg	This work
*evpB* Check_R	ctgctgctgaaactgctgag	This work
*impK* Check_F:	gctggatatgcacagtgacg	This work
*impK* Check_R:	aaacactgaccacagcacca	This work
*icmF* Check_F:	cagcagtaccggatgctctt	This work
*icmF* Check_R:	cagtttccagttcagctccg	This work
	**Gene complementation**	
*hcp* _Fw_*XbaI*	gcg tctaga gatggctattcctgcttatct	This work
*hcp* _Rv_*HindIII*	gcg ttcgaa gctcaggcggaaggacgctcat	This work
*evpB* _Fw_*XbaI*	gcg tctaga gatgctgatgtctgtacaaca	This work
*evpB* _Rv_*HindIII*	gcg ttcgaa gctcaggctttcgctttcggca	This work
*impK* _Fw_*XbaI*	gcg tctaga gatgaaaaaagatatggatat	This work
*impK* _Rv_*HindIII*	gcg ttcgaa gcttaacgcaggctttgcggca	This work
*icmF* _Fw_*XbaI*	gcg tctaga ggtgttcaaatttcccacatc	This work
*icmF* _Rv_*HindIII*	gcg ttcgaa gcttaatacaacgtatccggta	This work
	**Check insertion in pBAD24**	
pBAD24_Fw	atgccatagcatttttatcc	Guzman et al. (1995)
pBAD24_Rv:	gatttaatctgtatcagg	Guzman et al. (1995)	
	**qRT-PCR**	
qRT_*hcp* _F	gatgcctccagcccgtatct	This work
qRT_*hcp* _R	cacttcctgaccggcatcgt	This work
qRT_*evpB* _F	aaacgcctcgttcgcctttg	This work
qRT_*evpB* _R	tggatgggcagatcggctac	This work
qRT_*impK* _F	tttcgcgggcgttatcagga	This work
qRT_*impK* _R	gggtgctgacgggtggataa	This work
qRT_*vasK* _F	gactcgctctccggcattct	This work
qRT_*vasK* _R	gtgtccgtcagcgcaatcag	This work
qRT_*icmF* _F	gcagcgttatctcccctcgt	This work
qRT_*icmF* _R	ctcgttgttgcgcccacttt	This work

**Notes.**

a Forward primer extension to amplify chloramphenicol resistance cassette (tgtaggctggagctgcttcg).

b Reverse primer extension to amplify chloramphenicol resistance cassette (atgggaattagccatggtcc).

PCR products were subjected to horizontal gel electrophoresis in a 1.5% agarose gel (LE Agarose, Lonza, Alpharetta, GA) at 200 V for 70 min. A Hi-Lo molecular weight marker (50–10,000bp; Minnesota Molecular, Minneapolis, MN) and negative (sterile water) and positive controls from our lab collections were included as necessary. After electrophoresis, the gel was stained in 0.25% ethidium bromide solution (Sigma Aldrich. St. Louis, MO) for 20 min and viewed under UV light using an Omega Lum G imager (Aplegen, San Francisco, CA).

### Construction of mutants and complemented strains

Isogenic mutants were constructed for the T6SS genes *hcp*, *evpB* and *impK* from T6SS cluster 1 (T6SS1) and *icmF* from T6SS cluster 2 (T6SS2) using the Lambda-Red recombination system (Datsenko & Wanner, 2000) using the APECO18 as parental strain. Briefly, oligonucleotides specific to the chloramphenicol cassette flanked by 50 nt extensions homologous to 5′- and 3′-ends of the gene to be deleted were used to amplify the chloramphenicol resistance cassette from plasmid pKD3 (ATCC^®^, Manassas, VA). The PCR products were run on a 1.5% agarose gel, and gel extraction of the specific fragment was performed using QIAquick Gel Extraction Kit (Qiagen, Germantown, MD). The extracted fragments were electroporated into APECO18 containing the lambda-red expression plasmid pKD46 (ATCC^®^, Manassas, VA). After electroporation, the cells were grown in SOC (super optimal broth with catabolite repression) for 90 min and plated on LB agar containing 25 µg/mL chloramphenicol. Colonies were screened by PCR to identify deletion mutants. The chloramphenicol resistance cassette was cured by transforming the helper plasmid pCP20 (ATCC^®^, Manassas, VA) into the mutants and screening for chloramphenicol sensitive colonies. *In trans* complementation was performed by cloning PCR-amplified genes into the *XbaI* and *HindIII* restriction sites of plasmid pBAD24 (ATCC^®^, Manassas, VA), and transforming the construct into their mutant counterparts. Primers used are listed in Table 2.

### Growth curve analysis

The growth of WT APECO18 and mutant strains was analyzed in minimal medium M9 broth. Briefly, strains were incubated overnight in LB broth containing chloramphenicol (10 µg/mL) at 37 °C. Next, OD_600_ of the cultures was measured and cultures were diluted to an OD_600_ of 0.05 in M9 supplemented or not with arabinose at a final concentration of 0.2%. Cultures were incubated at 37  °C with shaking at 220 rpm, and OD _600_ measurements were obtained every 30 min. To measure OD_600_, 300 µL of the growing culture was dispensed in a well of a 96 well plate, and the absorbance was read using an ELX 808 Ultra microplate reader (Bio-Tek Instruments, Winooski, VT). Growth curves were performed in biological duplicates. Absorbance of replicates was averaged, and data was plotted against time to build the growth curves. Growth curves were carried out for a total of 7–8 h.

### Analysis of Expression of T6SS genes

#### RNA extraction

To assess the expression of *hcp*, *evpB*, *impK*, *icmF* and *vasK* genes, APECO18 was grown overnight at 37 °C statically. Next day, the cultures were diluted 1:100 in either LB broth or DMEM (ATCC^®^, Manassas, VA) and incubated at either 37 °C or 42 °C with shaking and grown to exponential phase (OD_600_ ∼0.6). RNA from biological duplicate cultures was extracted using the RiboPure™ RNA purification kit (Ambion, Austin, TX) according to manufacturer’s instructions. The RNA of APECO18 was also extracted after two hours of contact with DF-1 chicken fibroblasts to test whether cellular contact induced the expression of T6SS genes. Isolated RNA was treated with DNase 1 according to the manufacturer’s instructions to eliminate DNA trace amounts from the eluted RNA. The concentration of RNA samples was determined using a NanoPhotometer^®^ NP80 (Implen, Munchen, Germany), and the samples were stored at −80 ° C until use.

### qRT-PCR analysis

DNase-treated RNA was reverse transcribed using the First-Strand cDNA synthesis Kit from APExBio (Boston, MA). Briefly, 1 µg of DNAase-treated RNA was mixed with 1 µL of Random primers (50 µM), 1 µL of 10mM dNTP mixture, and adjusted to 10 µL with RNase-free water. The mixture was heated at 65 °C for 5 min and chilled on ice for 2 min for denaturation. The mixture was then centrifuged, and the cDNA synthesis mix was prepared by adding 4 µL of 5x first-strand buffer, 1 µL of RNase inhibitor, 1 µL of Reverse Transcriptase and RNase-free water to a total volume of 20 µL. The cDNA synthesis reaction was set as follows: 2 min at 25 °C, 50 min at 42 °C, and 15 min at 75 °C. The resultant cDNA was diluted 1:20 before use as template for qRT-PCR and stored at −20  °C until use.

Quantitative real-time RT-PCR (qRTPCR) was performed in a qTower^3^ G qPCR System (Analytik Jena, Jena, Germany) and analyzed using qPCRsoft v 4.1 software. Primers for qRT-PCR were obtained from Sigma Aldrich (St. Louis, MO). Reactions were performed using either qPCR Master Mix with Sybr^®^ Green (Goldbio, St. Louis, MO) or Platinum™ SYBR™ Green qPCR SuperMix-UDG (Invitrogen, Carlsbad, CA). Each reaction was performed in a final volume of 20 µl containing 25 ng cDNA (1 µL diluted cDNA), 1 µL each primer (10 µM), 10 µL 2x qPCR Master Mix Sybr^®^ Green (Goldbio, St. Louis, MO) or 10 µL Platinum™SYBR™ Green qPCR SuperMix-UDG (Invitrogen, Carlsbad, CA) and nuclease-free water to a final volume of 20 µL. When using Platinum™ SYBR™ Green qPCR SuperMix-UDG was used 1 µL of (1:10) ROX Reference Dye was added to each reaction. PCR conditions were as follows: 50  °C for 2 min hold, 95 °C for 2 min hold, and 40 cycles of 95 °C for 15 s and 60  °C for 30 s.

Threshold fluorescence was established within the geometric phase of exponential amplification, and the cycle threshold (CT) was determined for each RNA sample. Experiments were performed in biological and technical duplicates. The CT from each replicate was averaged. Expression levels were normalized using the housekeeping gene 16S rRNA as endogenous control. Melting curve analyses were performed after each reaction to ensure amplification specificity. Differences (n-fold) in transcripts were calculated using the relative comparison method (Schmittgen et al., 2000).

### Adherence and invasion assays

Cell adherence and invasion assays using chicken fibroblasts DF-1 cells were performed as previously described (Barbieri et al., 2017) with slight modifications. The cells were cultured in 75 cm ^2^cell culture flasks (Corning^®^, NY) in DMEM-F12 (ATCC^®^, Manassas, VA) containing 10% fetal bovine serum (FBS; Sigma, MO) with incubation at 37 °C and 5% CO_2_. Cells were transferred to sterile 24-well-plates at a concentration of 1 × 10^5^ cells/well 48 h prior each experiment. Wild-type APECO18, mutants and complemented strains were grown statically in LB broth overnight. Next, cultures were diluted 1:50 in fresh LB broth and grown at 37 °C with shaking at 220 rpm for 2 h. Cultures were then induced with 0.2% L-arabinose for 2 h. After induction, cultures were washed with PBS and adjusted to 1 ×10^8^cells/mL. 10 µL of bacterial suspension was used for infection. DF-1 cells were washed once with PBS and then exposed to bacteria at a multiplicity of infection (MOI) of 10. The 24-well-plates were centrifuged at 500 ×g for 5 min and incubated for 1 h for adherence and 4 h for invasion. The following steps were performed as described by (Barbieri et al., 2017)

The input dilution of bacteria was also plated and counted to determine the CFU for each inoculum used in the assay. Each experiment was performed in technical triplicates. The data presented here represents the average of two different experiments.

To visualize the association between bacteria and DF-1, 1 ×10 ^5^ cells per well were plated on glass coverslips in 24-well-plates and infected with a MOI of 10 CFU/cell as described above. After 1 h or 4 h post-infection, cells were fixed with 3.7% formaldehyde in PBS for 10 min at room temperature and stained with Giemsa for 20 min at room temperature. Samples were then washed with water and observed under a light microscope at 1,000x and photographed.

### Plaque assay

The plaque assay was performed as previously described (Hasselbring, Patel & Schell, 2011) with slight modifications. Overnight cultures of wild type APECO18, mutant and complemented strains were diluted 1:50 and grown to exponential phase at 37 °C with shaking. Then 0.2% L-arabinose was added to the cultures of the complemented strains for induction of gene expression, with incubation for an additional 2 h. Bacteria were then pelleted by centrifugation at 5,700 ×*g* for 7 min, washed with SorC (16.7 mM Na_2_H/KH_2_PO_4_/50µM CaCl_2_, pH 6.0) and re-suspended in SorC to 5 ×10^7^cFU/mL.

*D. discoideum* AX3 cells were cultured in HL5 broth and collected by centrifugation at 500 ×*g* for 5 min, washed once with SorC, re-suspended in SorC to 1 × 10^6^ cells/mL, and serially diluted in 10-fold increments in SorC. *D. discoideum* dilutions were mixed 1:1 with bacterial suspensions to generate bacterium-to-amoeba ratios ranging from 5 ×10^1^:1 to 5 ×10^5^:1. Aliquots of 10 µL of the mixed suspensions were spotted onto SM/5 agar plates and allowed to dry in a laminar hood under a sterile airflow. The plates were incubated at 22 ° C, examined after 3 and 5 d of incubation for plaque formation by *D. discoideum*, and photographed. Strains were then classified as sensitive or resistant to predation by *D. discoideum* based on the presence or absence of “predation plaques” formed by the amoeba. Experiments were done in duplicate.

### Statistical analysis

For the analysis of the prevalence of T6SS genes harbored by strains from different collections examined in the study, the number of genes were treated as quantitative variables and the data was analyzed using non-parametric tests due to asymmetry in the distribution of the genes. Non-parametric analysis was also applied to compare the phenotypes presented by the mutants in comparison to the WT strains. Direct comparisons (where possible) between two groups were made using the Mann–Whitney *U* test. All statistical analysis was performed using GraphPad Prism (Version 7.0d) for Windows (GraphPad, La Jolla, CA). Statistical significance was accepted when *p* < 0.05. For adherence and invasion assays, bacterial groups were compared using Student’s *t-* test and differences were considered significant when *p* < 0.05.

## Results

### Prevalence of T6SS genes is significantly higher in APEC than in poultry litter *E. coli* and AFEC isolates

**Figure 1:**
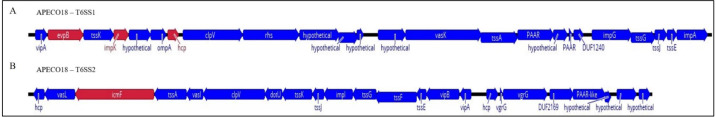
Schematic diagram of APECO18 T6SS gene clusters 1 (T6SS1, A) and 2 (T6SS2, B). The direction of the arrows indicates the direction of transcription. Arrows colored in red indicate the genes that were individually deleted from the APECO18 genome.

T6SS clusters are common in the genomes of *E. coli* pathotypes and are divided into three groups based on size, homology and structural analysis of clusters (Ma et al., 2014; Ma et al., 2013). Here, genomic analysis found that APECO18 harbors two putative T6SS clusters, T6SS1 and T6SS2. The T6SS1 cluster is 30.2 kb in length, with a GC content of 52.2%, and is flanked by tRNA genes. The T6SS2 cluster is 27.9 kb in length, with a 52% GC content and also flanked by tRNA genes. Most of the genes analyzed in this study, namely *evpB*, *impK* and *hcp* belong to T6SS1 cluster. Additionally, we analyzed the *icmF* gene from cluster 2. Schematic representation of these clusters is shown in Fig. 1. To gain insight into the relationship between the presence of T6SS genes and the pathogenesis of APEC, we used PCR to screen a collection 454 APEC, 102 poultry litter *E. coli* and 106 AFEC isolates.

**Figure 2:**
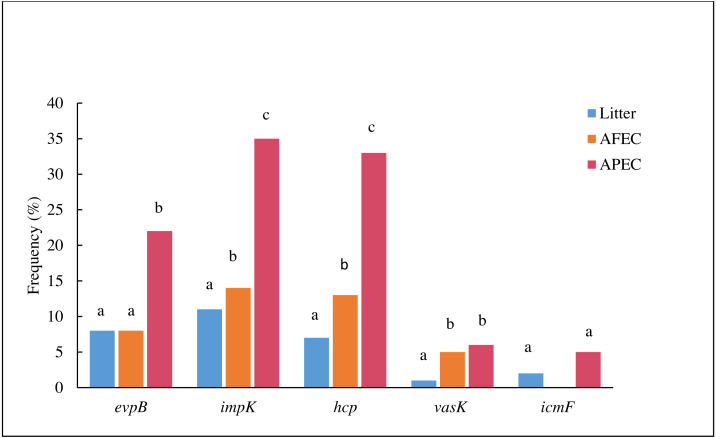
Histogram comparing the frequencies of genes *evpB*, *impK*, *hcp*, *vasK* and *icmF* in poultry litter *E. coli*, AFEC and APEC isolates. Different letters above bars indicate that the prevalence of the gene was significantly different between groups (*p* < 0.05).

The prevalence *evpB*, *impK* and *hcp* was significantly higher (*p* < 0.05) in APEC than in poultry litter *E. coli* and AFEC isolates; the prevalence of *impK* and *hcp* was also significantly higher in AFEC than in poultry litter *E. coli* isolates. The prevalence of *vasK* was significantly higher (*p* < 0.05) in APEC than in poultry litter *E. coli* isolates. *IcmF* was not detected in AFEC isolates, and the prevalence did not significantly differ between poultry litter *E. coli* and APEC isolates (Fig. 2). These findings support our hypothesis that T6SS contributes to the virulence of APEC.

### Deletion of T6SS genes did not affect the growth of APECO18 in minimal medium

To further investigate the role of T6SS genes in the pathogenesis of APEC strains, isogenic deletion mutants for *evpB*, *impK*, *hcp* and *icmF* were generated using APECO18 as the parental strain (Fig. S1). Prototypic strain APECO18, which belongs to O18 serotype and B2 phylogenetic group, was used as template for designing the primers. To exclude that the mutations have an influence on the growth rate of the mutants, APECO18 wild-type, mutants and complemented strains were assessed for their ability to grow in minimal medium M9. No differences were found in the growth rates of *hcp*, *evpB*, *impK* or *icmF* mutants compared to the wild-type strain in minimal medium (Fig. S2).

### APECO18 expresses genes encoding a T6SS

**Figure 3:**
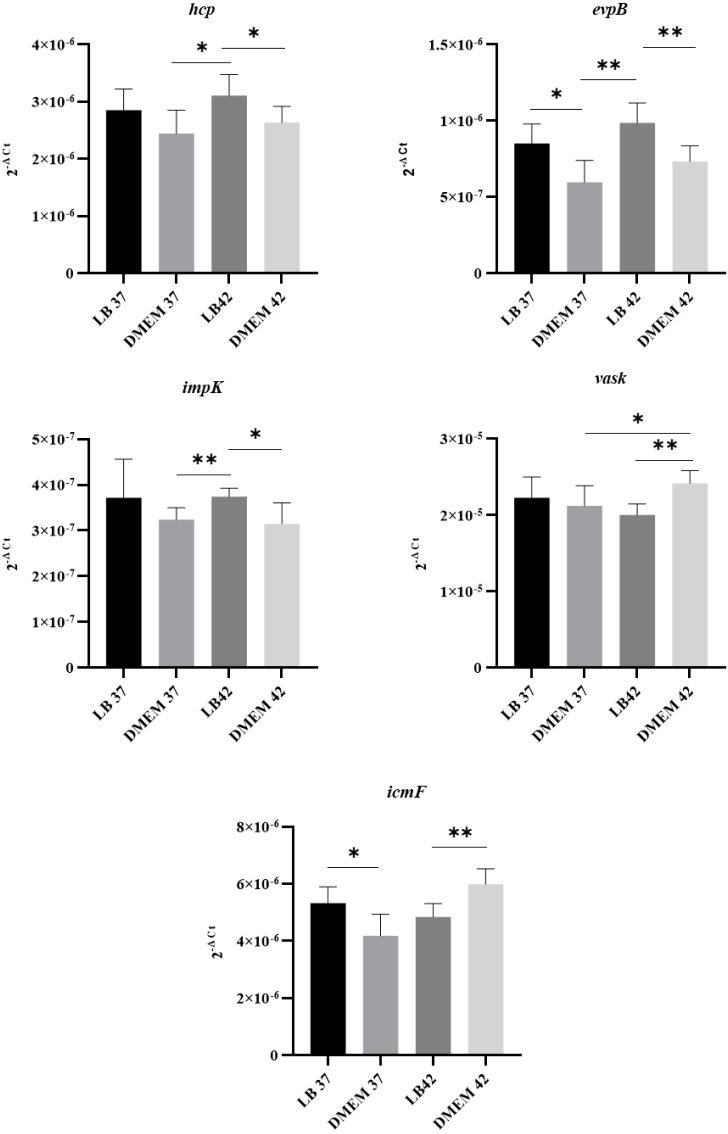
APECO18 expresses genes encoding a T6SS. qRT-PCR of *hcp*, *evpB*, *impK*, *vasK* and *icmF* genes by APECO18 in LB and DMEM at 37 °C and 42 °C. *, *p* < 0.05.

Since T6SS secretion has been shown to be temperature dependent in other organisms (Bladergroen, Badelt & Spaink, 2003), the expression of T6SS genes by APECO18 grown in LB and DMEM media at 37 °C or 42 °C was analyzed. Genes encoding 16S rRNA were used as endogenous controls for data normalization. As shown in Fig. 3, APECO18 expressed T6SS genes in both LB and DMEM with significant differences (*p* < 0.05) observed according to the media or temperature used. For instance, *evpB* expression was significantly higher (*p* < 0.05) in LB regardless of the temperature; *vasK* expression was significantly higher (*p* < 0.05) in DMEM at 42 °C compared to DMEM 37 °C and compared to LB at 42 °C.

**Figure 4:**
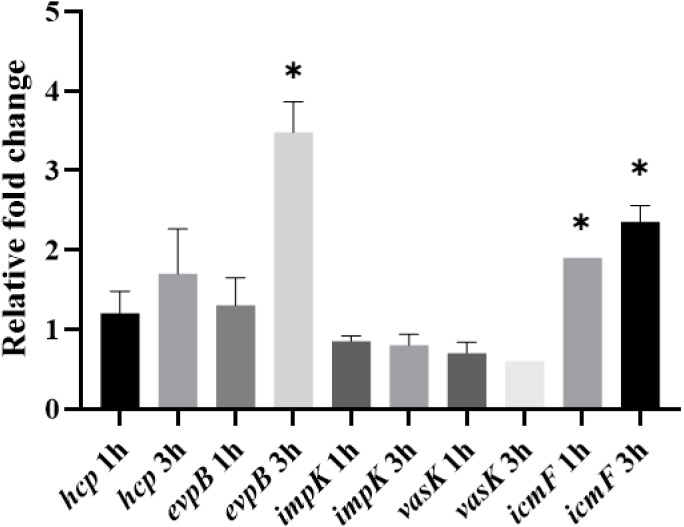
Expression of T6SS genes by APECO18 is enhanced post-infection of DF-1 cells. Expression of *evpB* enhanced at 3 h post infection, and expression of *icmF* is enhanced at 1h and 3 h post infection. Fold change is relative to APECO18 grown in DMEM at 37 °C. *, *p* < 0.05.

Because expression of the T6SS has been shown to be upregulated in the presence of the host (De Pace et al., 2010), we also assessed whether the expression of T6SS genes by APEC O18 was upregulated in the presence of chicken fibroblasts DF-1. Expression of *evpB* was significantly (*p* <  0.05) enhanced at 3 h post-infection, and expression of *icmF* was significantly enhanced (*p* < 0.05) at 1 h post-infection and enhanced further at 3 h post- infection (Fig. 4).

### Role of T6SS in the adherence of APECO18 to chicken fibroblasts DF-1

Because T6SS genes *icmF*, *hcp*, and *clpV* have been previously recognized as being involved in the binding of APEC Sept362 to HeLa cells (De Pace et al., 2011; De Pace et al., 2010), we assessed whether *hcp* and other T6SS genes (*evpB*, *impK*, and *icmF*) are involved in the adherence of APECO18 to chicken fibroblasts DF-1.

**Figure 5:**
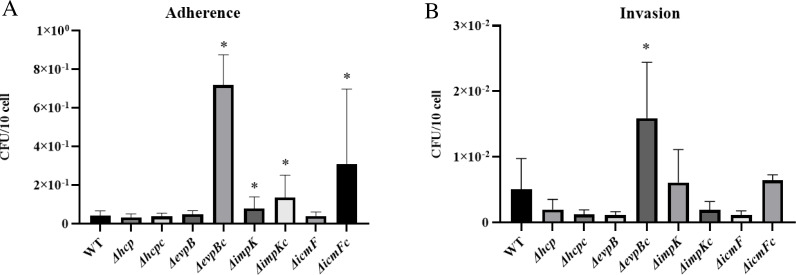
Role of T6SS in adherence to and invasion of DF-1 chicken fibroblasts by APECO18. (A) Quantification of adherence rate of APECO18 wild-type, mutant and complemented strains to DF-1. (B) Quantification of invasion rate of DF-1 by APECO18 wild-type, mutant and complemented strains (*, *p* < 0.05).

We did not observe a negative effect of the mutation of T6SS genes *hcp*, *evpB*, *impK*, and *icmF* in the adherence capability of APECO18. An unexpected significant increase (*p* < 0.05) of 90% in the adherence APECO18 Δ*impK* in relation to the WT strain was observed. Complemented strains APECO18 Δ*impK*c, APECO18 Δ*evpB*c and APECO18 Δ*icmF*c showed an adherence rate that was 2.27, 16.30 and 6.47 times higher than the wild-type respectively (*p* < 0.05) (Fig. 5A). These results suggest that the T6SS genes analyzed in this study are not involved in the adhesion of APECO18 to chicken fibroblasts DF-1.

### Role of T6SS in the invasion of chicken fibroblasts DF-1 by APECO18

Deletion of *hcp* and *icmF* has been shown to affect the invasion of HeLa cells by APEC Sept362 (De Pace et al., 2011; De Pace et al., 2010). To investigate whether *hcp*, *evpB*, *impK* and *icmF* are involved in invasion of chicken fibroblasts DF-1 by APECO18, we assessed the invasion capability of APECO18 WT, mutants and complemented strains.

Similar to what was found for adherence capability, we did not observe a negative effect of the mutation of T6SS genes on the invasion capability of the mutants compared to the wild-type strain. A decrease in invasion rate of 61%, 78% and 78% was observed for APECO18 Δ*hcp*, APECO18 Δ*evpB*, and APECO18 Δ*vasK* respectively in relation to the wild-type strain, however these differences were not statistically significant (*p*>0.05) (Fig. 5B). An unexpected increase of 20% in the invasion rate of DF-1 by APECO18 Δ*impK* compared to the WT strain was observed, but it was not significant (*p* >  0.05). Complemented strain APECO18 Δ*evpB*c showed an invasion that was 3.13 times the invasion rate presented by the wild-type APECO18 (*p* < 0.05).

### Deletion of *hcp* increases sensitivity of APECO18 to predation by *D. discoideum*

**Figure 6:**
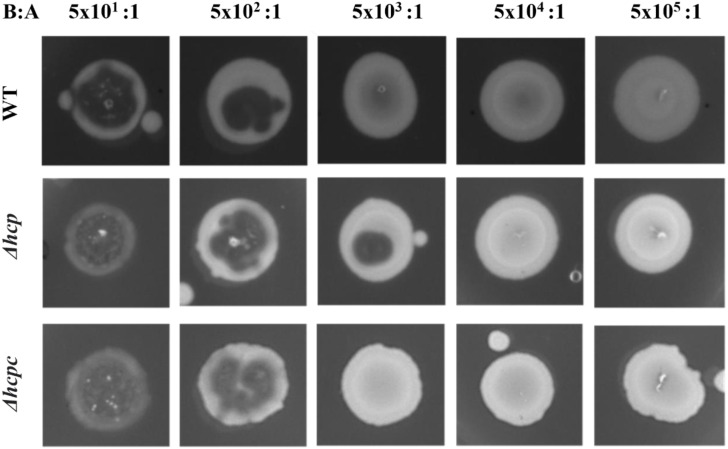
Deletion of *hcp* increases sensitivity of APECO18 to predation by *D. discoideum*. Figure shows predation plaque assay for wild-type, mutant (Δ*hcp*) and complemented strain (Δ*hcpc*) at different bacteria-to amoeba ratios (B:A).

We assessed the ability of APECO18 WT, mutants and complemented strains to resist to predation by *D. discoideum.* Deletion of *impK* and *icmF* did not affect resistance of APECO18 to predation by *D. discoideum*. An increased sensitivity was observed in the Δ*evpB* strain but no restoration was observed in the complemented strain indicating a possible polar effect of the *evpB* deletion on this phenotype (Fig. S3). However, deletion of *hcp* affected the strain’s sensitivity to predation by *D. discoideum*. At the bacteria to amoeba ratio of 5 ×10^1^:1, the bacterial lawn was completely consumed and replaced by *D. discoideum* fruiting bodies in the WT, mutant, and complemented strain. At the ratio 5 ×10^2^:1 the amoeba consumed most of the bacteria, but some of the bacterial lawn was still observed at the edges of the spot in the WT, mutant, and complemented strains. The difference between the WT strain and Δ*hcp* was observed at the bacteria-amoeba ratio of 5 ×10^3^:1. At this ratio, the WT strain was resistant to predation by the amoeba, with no plaques observed. However, the mutant strain became sensitive to predation with a large predation plaque observed on the bacterial lawn. The phenotype was restored in the complemented strain. In summary, 500 amoeba cells were necessary to form a plaque on the WT bacterial lawn, while only 50 amoeba cells were able to form a plaque on the Δ*hcp* strain. This result indicates that Hcp is involved in the ability of APECO18 to resist predation by *D. discoideum* (Fig. 6).

## Discussion

APEC cause extra-intestinal infections in production birds known as colibacillosis, which can manifest as localized or systemic infections. The severity of the infection depends on the virulence traits of the strain, host status and predisposing factors (Barnes & Gross, 1997; Nolan et al., 2020). APEC has a diverse virulence trait repertoire, with a range of recognized virulence factors involved in its pathogenesis (Nolan et al., 2020). Although several virulence factors have been described so far, the pathogenesis of APEC strains remains unclear. Among its virulence genes, T6SS genes, *hcp*, *clpV* and *icmF* were shown to be involved in virulence-associated phenotypes displayed by APEC strain Sept 362 (De Pace et al., 2011; De Pace et al., 2010). Here, we analyzed the role of *hcp* and other T6SS components, *evpB*, *impK*, and *icmF* in virulence-associated traits of APECO18.

This study is also the first report to characterize a large collection of APEC, litter-associated *E. coli,* and fecal (AFEC) isolates for the presence of T6SS genes. The screening of these collections showed that T6SS genes are more prevalent in APEC than in fecal (AFEC) and litter-associated *E. coli* isolates, which led us to further characterize the T6SS in APECO18. Genomic analysis of APECO18 performed in this work found that APECO18 harbors two clusters encoding T6SSs. T6SS1, the larger cluster, is 30.2 kb in length, with a GC content of 52.2%, and is flanked by tRNA genes. T6SS2 is slightly smaller than T6SS1, with 27.9 kb in length, a 52% GC content and is also flanked by tRNA genes. T6SS2 is encoded on the complementary strand. These findings are in agreement with previous studies that showed that APEC strains can harbor up to three T6SS clusters, with 14.6% of the strains harboring T6SS1, 2.3% harboring T6SS2, and 0.8% harboring T6SS3, with most of the strains harboring two clusters and that they present in different sizes and orientations (Ma et al., 2013). The presence of clusters encoding two functional T6SS in APEC strain, TW-XM also corroborates our findings (Ma et al., 2014).

We generated mutants of *hcp*, *evpB*, *impK* (T6SS1), and *icmF* (T6SS2) in APECO18 and characterized these mutants for their potential impact on virulence-associated traits of APEC, including adherence to and invasion of DF-1 chicken fibroblasts, and resistance to predation by *D. discoideum*. We also evaluated the expression of these genes in bacterial culture and cell culture media and upon contact with chicken fibroblasts.

The expression of T6SS genes by APECO18 was analyzed under two different temperatures, as T6SS secretion has been shown to be temperature-dependent (Bladergroen, Badelt & Spaink, 2003). Our analysis of expression did not show a trend of expression according to temperature for all genes analyzed. Genes *hcp*, *evpB* presented higher expression at 42°C in both LB and DMEM while the highest expression of *vasK* and *icmF* were observed in DMEM 42 °C. *ImpK* on the other hand showed greater expression in LB than in DMEM. In another study that analyzed the expression of T6SS genes by *Francisella noatunensis* subsp. *orientalis* at different temperatures, the authors noted a trend of higher expression of the genes at lower temperature (25 °C) with the exception of *pdpB*, which showed higher expression at 30 ° C (Lewis & Soto, 2019). These observations corroborate with our data showing that not all the genes follow the same trend of expression at different temperatures.

Following confirmation of expression of T6SS genes in bacterial culture and cell culture media, we evaluated whether contact with chicken fibroblast DF-1 cells would influence upregulation of the expression of T6SS genes. We found that expression of *evpB* and *icmF* by APECO18 was upregulated upon contact with the host cells, in a manner somewhat similar to what was found for the expression of *hcp* and *clp* by APEC SEPT362 (De Pace et al., 2010). Adherence of bacteria to host cells is important during the initial stages of colonization and is usually the first step in infection caused by *E. coli* (Antão, Wieler & Ewers, 2009). The T6SS has been previously associated with the adherence of APEC to their host cells. In Sept362, mutation of the T6SS genes *hcp*, *clpV* and *icmF* led to a significant reduction of the adherence of the strain to HeLa cells (De Pace et al., 2011; De Pace et al., 2010). We did not observe a significant negative effect of the mutation of T6SS genes in the adherence capability of APECO18. In contrast, complemented strains showed a significantly higher adherence rate when compared to the wild-type.

T6SS genes have also been found to play a role in invasion of the host cell model by APEC and other *E. coli*. In Sept362, deletion of *hcp* and *icmF* significantly decreased the ability of the strain to invade HeLa cells (De Pace et al., 2011; De Pace et al., 2010). In the present work, we did not observe any negative effect associated with the deletion of *hcp*, *evpB*, *impK* or *icmF* in the ability of APECO18 to invade chicken fibroblasts DF-1 - a model cell line for poultry. These observations contrast with those of de Pace and colleagues suggesting that the cell model used may also be an influencing factor on APEC invasion and warrants further investigation. In addition, other systems also impact the invasive capability of APEC and likely compensate for the lack of T6SS genes analyzed here.

We have also analyzed the resistance of wild-type and mutants to predation by the amoeba *D. discoideum* cell model, that feeds on bacteria through well-characterized phagosomal and endolysosomal mechanisms similar to those of mammalian phagocytes (Buczynski et al., 1997; DeShazer, Brett & Woods, 1998; Maniak et al., 1995; Rezabek et al., 1997; Rupper &cardelli, 2001). Studies of bacterial pathogens have found that genes required for resistance to predation by *D. discoideum* are also involved in the replication or survival of bacteria in mammalian macrophages (Andrews, Vogel & Isberg, 1998; Hägele et al., 2000; Solomon, Leung & Isberg, 2003; Solomon et al., 2000; Steinert & Heuner, 2005); and in some cases in causing disease in animals (Alibaud et al., 2008; Cosson et al., 2002; Finck-Barbançon et al., 1997; Hauser, Kang & Engel, 1998; Pearson et al., 2000; Pukatzki, Kessin & Mekalanos, 2002; Vance, Rietsch & Mekalanos, 2005). Additionally, the T6SS was implicated as a virulence determinant of *V. cholerae* using the *D. discoideum* model system (Pukatzki et al., 2006). Thus, we explored *D. discoideum* as a model for pathogenic interactions between APECO18 and macrophages. Results from our work show that deletion of *hcp* led to increased sensitivity of the strain to the predation by *D. discoideum*. Hcp functions as a structural component of T6SS that forms a transportation channel between the inner and outer membranes of the bacteria and a secreted effector injected into host cells (Pukatzki, McAuley & Miyata, 2009). Previous studies have shown that deletion of T6SS genes caused the strain to be avirulent towards *D. discoideum.* In *V. cholerae*, deletion of T6SS genes VCA0109 through VCA0114 and VCA0119 made the strain avirulent towards the amoeba, with 10,000 amoeba required to form a predation plaque on the WT lawn, while only 5 amoeba were required to form a plaque on the mutant strain of *V. cholerae* O37 strain V52 (Zheng, Ho & Mekalanos, 2011). Here, we show that 500 amoeba cells were necessary to form a plaque on the WT bacterial lawn, while only 50 amoeba cells were able to form a plaque on the Δ*hcp* strain, indicating a higher sensitivity of this mutant to the predation by *D. discoideum* in comparison to the WT strain.

## Conclusion

The T6SS plays a role in APEC pathogenesis as evidenced by its prevalence in APEC compared with AFEC isolates and poultry litter associated *E. coli*, its involvement in the ability of APECO18 to resist predation by *D. discoideum* and in its ability to adhere and invade chicken fibroblasts and expression. Further work including *in vivo* and *in vitro* assays to dissect the role of T6SS on the virulence of APEC and its effects on the poultry host is warranted.

##  Supplemental Information

10.7717/peerj.12631/supp-1 Supplemental Information 1 Construction of T6SS mutants in APECO18 M –size marker (Hi-Lo molecular weight marker). Lane 1: WT *hcp*; Lane 2: Δ *hcp*; Lane 3: WT *evpB*; Lane 4: Δ *evpB*; Lane 5: WT *impK*; Lane 6: Δ *impK*; Lane 7: WT *icmF*; Lane 8: Δ *icmF*.

10.7717/peerj.12631/supp-2 Supplemental Information 2 Deletion of T6SS genes does not affect the growth of APECO18 in minimal media Growth curves of (A) APECO18 WT, Δ *hcp*, Δ *hcp* c, (B) APECO18 WT, Δ*evpB*, Δ *evpB* c, (C) APECO18 WT, Δ *impK*, Δ *impK* c, (D) APECO18 WT, Δ *icmF*, Δ *icmF* c at 37 °C. The expression of the genes in the complemented strains was induced with 1.5 mM L-arabinose. Experiments were performed in biological duplicates. Error bars represent standard deviation.

10.7717/peerj.12631/supp-3 Supplemental Information 3 Plaque assay for WT APECO18, Δ*icmF,*Δ*evpB,*Δ*impK* and complemented strains at different bacteria-to-amoeba ratios (B:A) Deletion of *impK* and *icmF* did not affect resistant of APECO18 to predation by *D. discoideum*. An increased sensitivity was observed in the Δ*evpB* strain but no restoration was observed in the complemented strain indicating a possible polar effect of *evpB* deletion on this phenotype.

10.7717/peerj.12631/supp-4 Supplemental Information 4 T6SS data on isolates screened

## Additional Information and Declarations

### Data Availability

The following information was supplied regarding data availability: The raw data is available in the Supplementary Files.
