# Supplementary figures and images for: Characterizing the Type 6 Secretion System (T6SS) and its role in the virulence of avian pathogenic Escherichia coli strain APECO18
Source: PeerJ. 2021 Dec 17;9:e12631. doi: 10.7717/peerj.12631 (PMC8686734; doi:10.7717/peerj.12631)

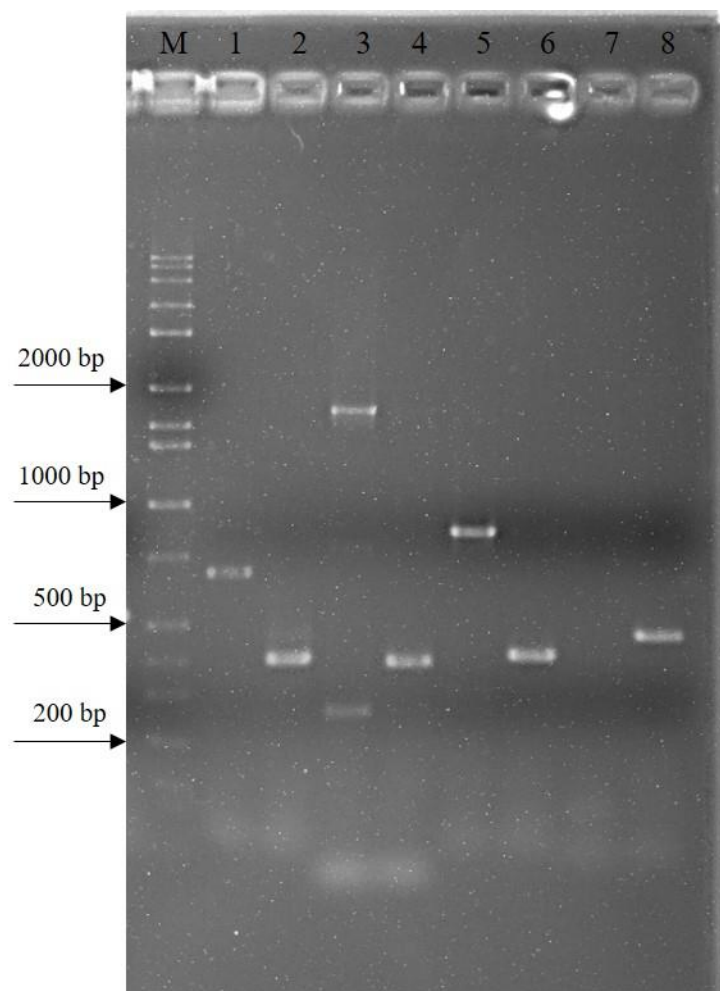

Supplement: Supplemental Information 1 — M –size marker (Hi-Lo molecular weight marker). Lane 1: WT hcp; Lane 2: Δ hcp; Lane 3: WT evpB; Lane 4: Δ evpB; Lane 5: WT impK; Lane 6: Δ impK; Lane 7: WT icmF; Lane 8: Δ icmF. [file peerj-09-12631-s001.pdf]

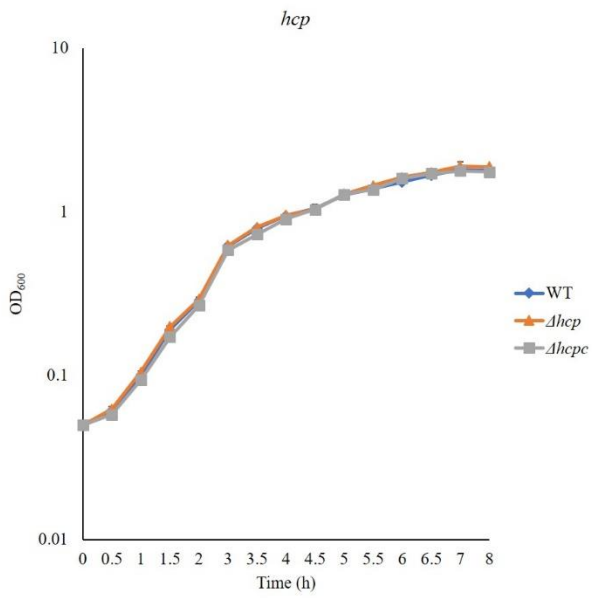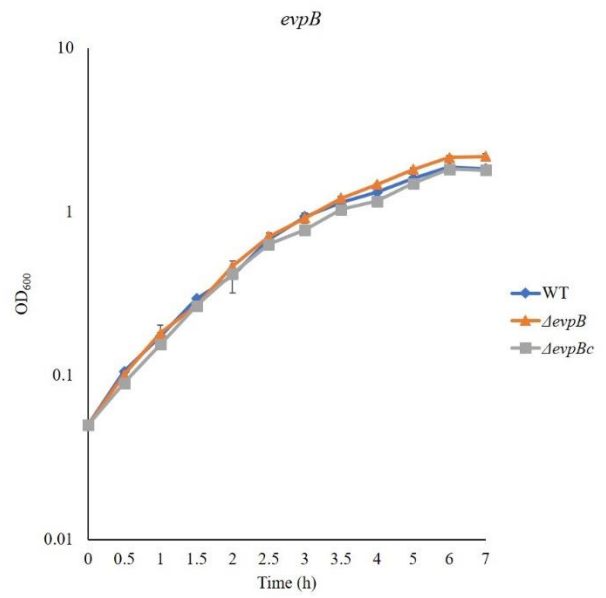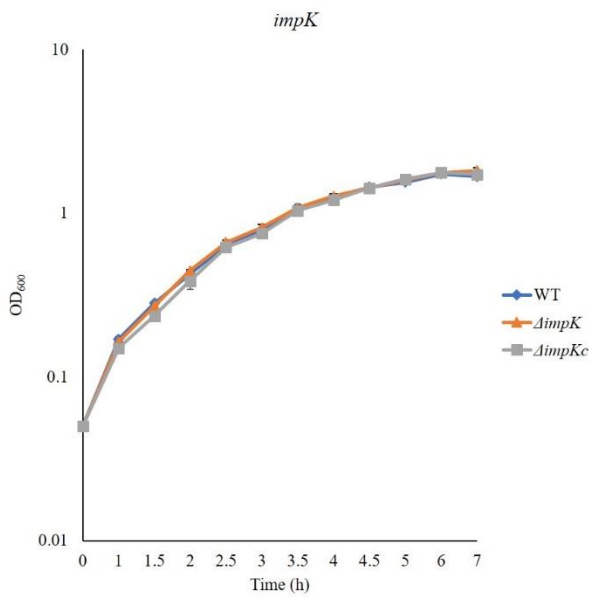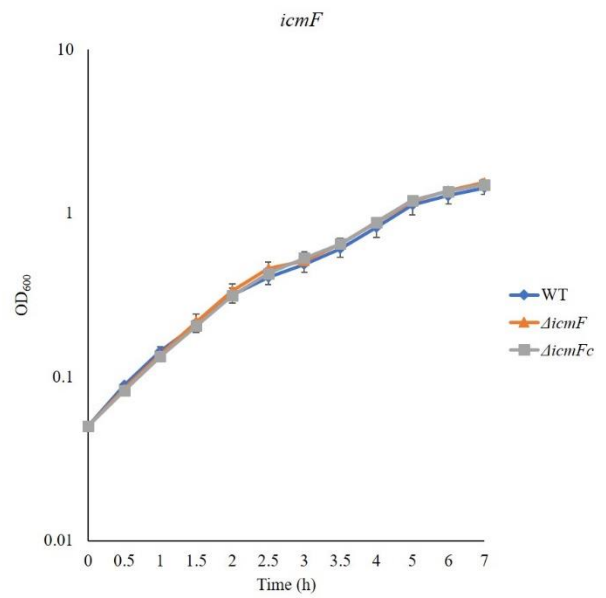

Supplement: Supplemental Information 2 — Growth curves of (A) APECO18 WT, Δ hcp, Δ hcp c, (B) APECO18 WT, ΔevpB, Δ evpB c, (C) APECO18 WT, Δ impK, Δ impK c, (D) APECO18 WT, Δ icmF, Δ icmF c at 37 °C. The expression of the genes in the complemented strains was induced with 1.5 mM L-arabinose. Experiments were performed in biological duplicates. Error bars represent standard deviation. [file peerj-09-12631-s002.pdf]

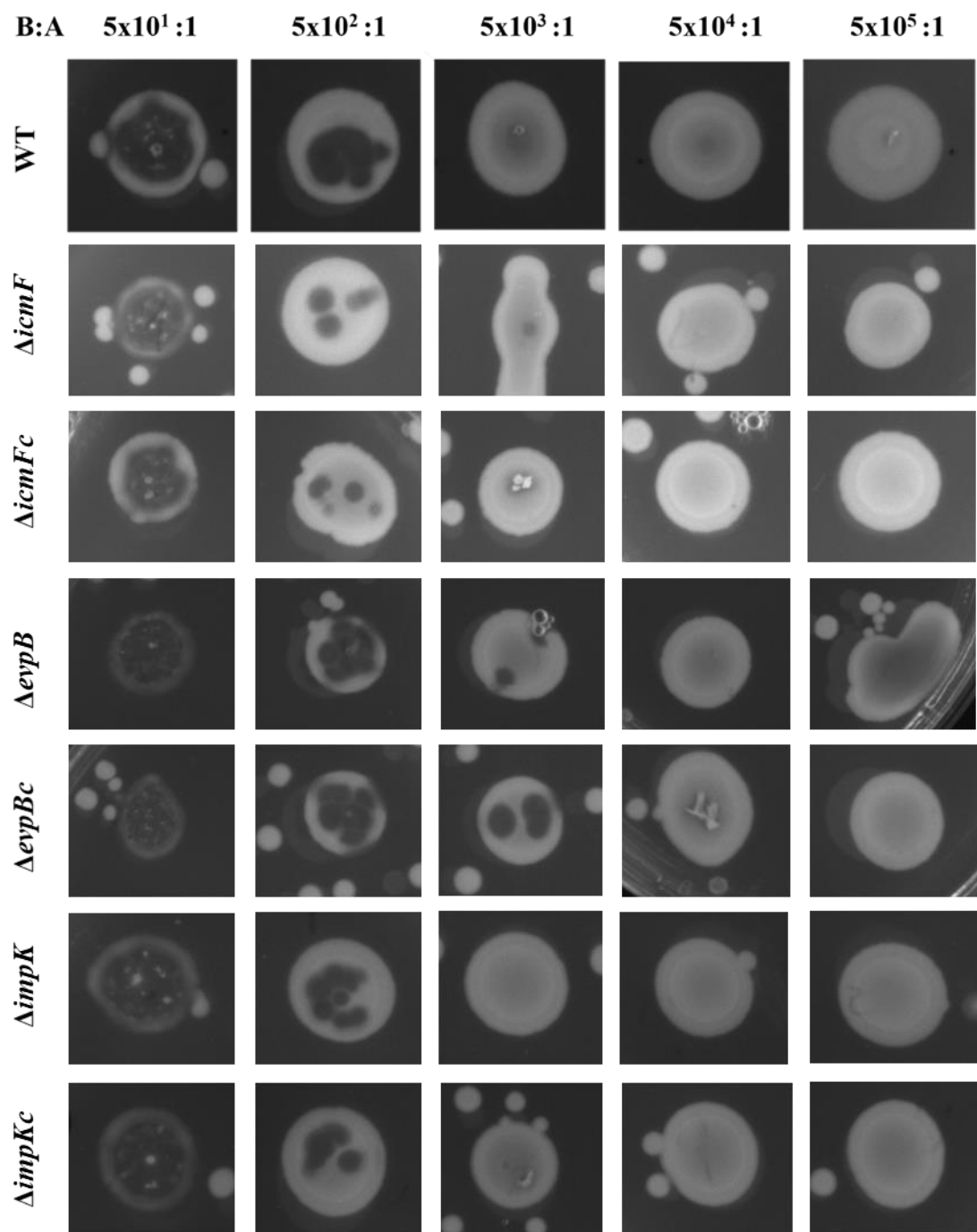

Supplement: Supplemental Information 3 — Deletion of impK and icmF did not affect resistant of APECO18 to predation by D. discoideum. An increased sensitivity was observed in the ΔevpB strain but no restoration was observed in the complemented strain indicating a possible polar effect of evpB deletion on this phenotype. [file peerj-09-12631-s003.pdf]
